# Protocol for constructing a hierarchical host-guest supramolecular self-assembly system in water

**DOI:** 10.1016/j.xpro.2023.102488

**Published:** 2023-07-28

**Authors:** Xiao Chen, Xu-Man Chen, Quan Li

**Affiliations:** 1 Institute of Advanced Materials and School of Chemistry and Chemical Engineering, Southeast University, Nanjing, 211189, China; 2 Materials Science Graduate Program, Kent State University, Kent, OH 44242, USA

**Keywords:** Microscopy, NMR, Chemistry, Material Sciences

## Abstract

Here, we present a protocol for the construction of a hierarchical host-guest supramolecular self-assembly system in water. We describe steps for determining the binding levels and capturing the morphologies of hierarchical self-assembly. We detail procedures for using UV-vis spectra, nuclear magnetic resonance spectra, scanning electron microscopy, and transmission electron microscopy for the assembly. This protocol is useful for analyzing the detailed chemical structure and morphological variation of hierarchical host-guest supramolecular self-assembly systems.

For complete details on the use and execution of this protocol, please refer to Chen et al. (2022).^1^

## Highlights

• Detailed protocol for determining the binding levels of hierarchical self-assemblies
• Detailed protocol for molecular binding structures of hierarchical self-assembly by NMR
• Detailed protocol for capturing the morphologies of hierarchical self-assemblies

## Abstract (editor highlights)

Publisher’s note: Undertaking any experimental protocol requires adherence to local institutional guidelines for laboratory safety and ethics.

## Graphical abstract

**Figure:**
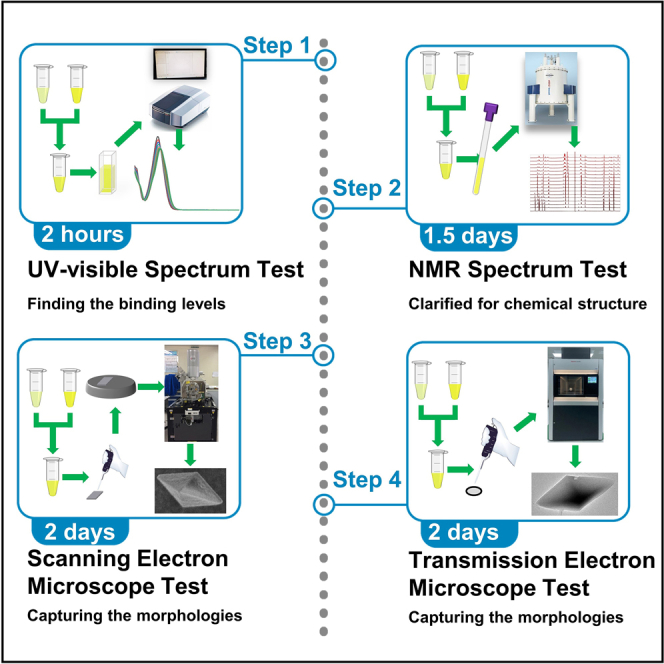

## Before you begin

This protocol describes the detailed steps for identifying a hierarchical host-guest supramolecular self-assembly system in water via UV-vis spectra, NMR spectra, scanning electron microscopy (SEM), and transmission electron microscopy (TEM) according to the publication from Chen et al.^1^ Therefore, the synthesized guest molecule 4,4’-(9H-carbazole-2,7-diyl)bis(1-butyl-pyridin-1-ium) dibromide (CPDB) and host molecule cucurbit[8]uril (CB[8]) are used as sample compounds for this protocol (detailed synthesis please refer to Chen et al.^1^). Before the determination of this self-assembly, a guest molecule aqueous solution, and a host molecule solution or dispersion (for some non-aqueous solution host molecule, like CB[8]) should be prepared. The water used for the preparation of sample for SEM and TEM tests should be filtered with microfiltration membrane (pore size: 220 nm) to avoid the possible interferences. But for UV-vis spectra test, this step is not necessary but recommended.

### Preparation of the aqueous solution of the guest molecule

**Timing: 1.5–2 h**

1. Preparation of deionized water in different pH that is regulated by diluted aqueous HCl and NaOH as test required.
  a. 1 M HCl solution is prepared by diluting concentrated HCl solution (12 M) with filtered deionized water.
  b. 1 M NaOH solution is prepared by dissolving required amount of NaOH solid with filtered deionized water.
  c. The deionized water is regulated to the required pH value.

***Note:*** If the pH value of water does not affect the test result, please just start test from step 2. For NMR tests, deuterated reagents such as D_2_O, DCl, NaOD are used for the preparation.

2. Preparation of aqueous solution of the guest molecule.
  a. The powder of the guest molecule is added into vessel, and further dissolved or dispersed with water prepared from step 1.
  b. The vessel with aqueous solution of the guest molecule is treated by ultrasound about 1 or 1.5 h to guarantee the complete dissolution of the guest molecule.

***Note:*** 1. If the guest molecule is poorly water-soluble, tiny amount of organic solvent can be used to help the dissolution of guest molecule in water. But the addition of organic solvent is suggested not exceeding 1% (v/v). 2. All vessels should be washed carefully by filtered deionized water and dried in a constant temperature (e.g., 25°C).

### Preparation of host molecule aqueous solutions or dispersions

**Timing: 1–3 h**

3. Preparation of aqueous solution of the host molecule.
  a. In a vessel, the host molecule is dissolved by deionized water that prepared from step 3.
  b. The vessel with aqueous solution of the host molecule is treated by ultrasound about 1 or 1.5 h to guarantee that the host molecule dissolves well.

***Note:*** if the host molecule is not well water-soluble, please see step 4.

4. Preparation of the host molecule aqueous dispersion with trace amount of guest molecule.
  a. In a vessel, the guest molecule (for introducing host molecule in water, and addition of guest molecule is often not inferior to 0.1 equivalent of host molecule) and host molecule are dispersed with water prepared from step 3.
  b. The vessel with the above-mentioned aqueous dispersion is put into ultrasound equipment about 2 or 2.5 h to make sure the sufficient dispersion of the host and guest molecules.

***Note:*** 1. If there is still insoluble solid in the sample, increase the temperature and prolong the ultrasonic time. 2. The host molecule aqueous solution or dispersion prepared in this step should be used as soon as possible to avoid the influence of precipitation on the experimental results.

**CRITICAL:** Hydrochloric acid is highly irritating and corrosive to eyes, skins, and the respiratory system. Handle it under a chemical fume hood, wear gloves, eye goggles and appropriate personal protective equipments.

**CRITICAL:** NaOH solid and NaOH aqueous solution is highly irritating and corrosive to eyes, skins, and the respiratory system. Handle it under a chemical hood wearing gloves and protective eye goggles.

## Key resources table

**Table:** 

REAGENT or RESOURCE	SOURCE	IDENTIFIER
**Chemicals, peptides, and recombinant proteins**		

HCl	Aladdin	H399545-500 mL
NaOH	Aladdin	S111518-500g
Deionized water	Aladdin	W119424-25L
D_2_O	Aladdin	D113903-10×1 mL
CPDB	Synthesized	
Cucurbit[8]uril	HWRK Chem	HWG46108

**Software and algorithms**		

UVProbe	Shimadzu	www.shimadzu.com
MestReNova	Mestrelab	www.mestrelab.com
Topspin	Bruker	www.bruker.com

**Other**		

Gilder 300 Mesh Square Grids	EMCN	AG300
Silicon wafer	EMCN	BZS0505
NMR tube	Synthware	XWE-5MM-7-50
Sample bottle	Synthware	V312250
CELL,10MM(S)	Shimadzu	200–34442
UV-vis spectrophotometer	Shimadzu	UV-2700
NMR instrument	Bruker	AVANCE III HD 600MHz
SEM	FEI	Navo Nano SEM450
TEM	FEI	Talos F200X

## Step-by-step method details

### UV-vis spectra

**Timing: 2–4 h**

**Figure 1:**
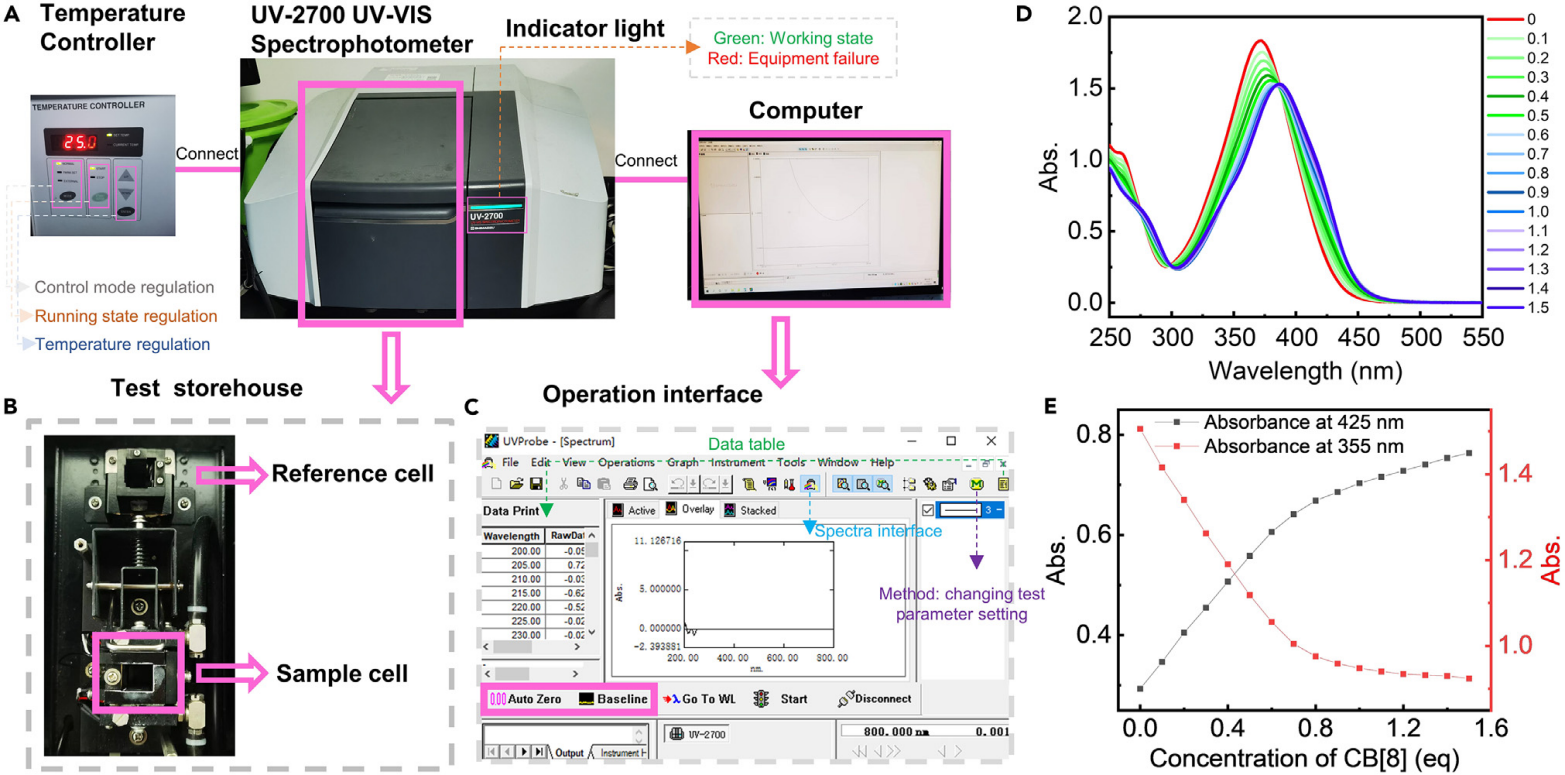
UV-vis spectra test (A) Illustration of UV-2700 UV-vis spectrophotometer with computer and temperature controller. (B) The image of test storehouse. The slot is used for holding sample cell and reference cell. (C) The image of operation interface for UV-2700. The key buttons are marked with pink squares. (D) UV-vis spectra of hierarchical host-guest supramolecular self-assemblies with CB[8] variation from 0 equiv. to 1.5 equiv. (E) The specific absorbance at 425 and 355 nm of hierarchical host-guest supramolecular self-assemblies with CB[8] variation from 0 equiv. to 1.5 equiv.

This section describes the variation of the UV-vis spectra of guest molecule by gradually adding host molecule, which can reveal changing of the assembly state in different binding levels. A UV-vis spectrophotometer system is required in this section (Figure 1A).

1. Dilute guest molecule aqueous solution according to the experimental requirements.

***Note:*** The recommended concentration of guest molecule in water is at least **4 × 10**^**–5**^**M**.

2. Setting parameters of the UVProbe software are as follows:

Measuring mode is absorbance;

Slit width is 2.0 nm;

Scan wavelengths range from 250 nm to 800 nm;

Scan speed is fast;

Sampling interval is 0.5 nm.

***Optional:*** Control the test temperature at a constant temperature (e.g., 25°C) according to experimental requirements (Figure 1A).

3. Two cells with background solvent are put into sample stage (Figure 1B), and “Base line” after “auto zero” is carried out by UVProbe software (Figure 1C).

***Note:*** The observed absorption value at 800 nm before each test should be within ±0.002.

**CRITICAL:** The sample cells are made by quartz, which may scratch skin when they are broken.

4. The test cell is replaced with guest molecule aqueous solution and click “start” to obtain the UV-vis spectra.
5. A certain and trace volume of the concentrated host molecule aqueous solution or dispersion is gradually added into test cell to guarantee the molar ratio of host and guest molecules reaching calculated valve.

***Note:*** For example, the molar ratio of guest and host molecule can reach from 1:0.1, 1:0.2, 1:0.3 … to 1:1.5

***Note:*** The UV-vis spectra are further obtained after the sample solutions are well blended.

***Note:*** Because our self-assembly system shows no absorption at 800 nm, the observed absorption value at 800 nm before each test should be within ±0.002.

6. The data table of UV-vis spectra can display on the screen by clicking “” on UVProbe software.
7. Draw the spectra according to the data table.
8. Analyze the spectra to find the absorption values at a specific wavelength and draw a plot of points by using these absorption values to find the variation tendency of UV-vis spectra for studying the different stages of hierarchical host-guest supramolecular self-assembly system, see Figures 1D and 1E.

### ^1^H-NMR

**Timing: 1–1.5 days**

**Figure 2:**
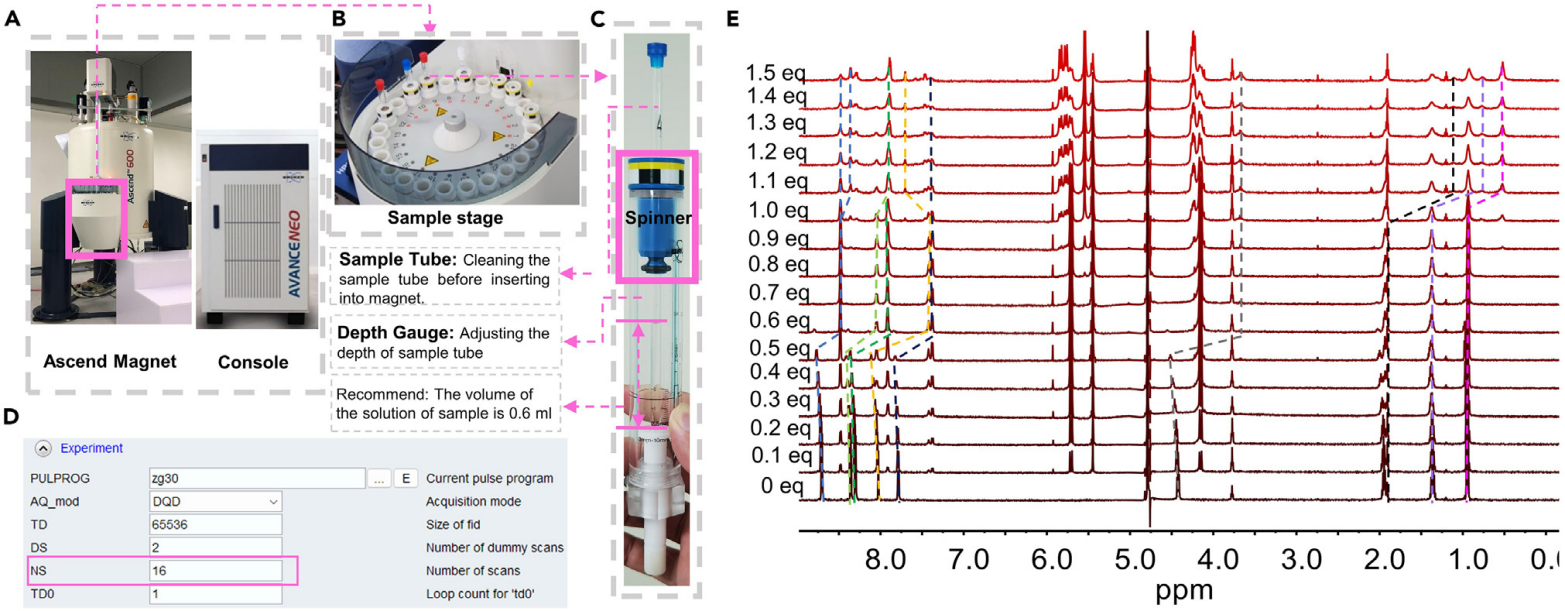
^1^H-NMR spectra test (A) Illustration of NMR instrument including magnets and consoles of Ascend. The pink square inside is the sample stage. (B) The image of the sample stage for holding NMR tube with spinner. This stage helps the test sample in line. (C) The image of NMR tube, spinner, and depth gauge. The function of spinner is rotating NMR tube in compressed air. The depth gauge is used for adjusting depth of NMR tube in Ascend magnets to avoid the collision and break of the NMR tube. (D) The parameter for NMR testing. Increasing the number of scans (NS) may make the peaks split more clearly. (E) The NMR spectra of hierarchical host-guest supramolecular self-assemblies with CB[8] variation from 0 equiv. to 1.5 equiv. The variation trends of the characteristic peaks are marked by dashed lines with different colors.

This section describes the variation of the guest molecule on ^1^H-NMR spectra by gradually adding host molecule, which also reveals the change of the hierarchical assembly states. An NMR instrument is required in this section (Figures 2A and 2B).

9. D_2_O solution of the guest molecule is prepared according to the step 2 of the Part: Preparation of the aqueous solution of the guest molecule after calculating the required volume of the D_2_O solution of the guest molecule.

***Note:*** The concentration of D_2_O solution of the guest molecule is suggested at least **1 × 10**^**–3**^**M** due to the sensitivity of ^1^H-NMR.

10. D_2_O solution of the host molecule is prepared according to the Part: Preparation of host molecule aqueous solutions or dispersions after calculating the required volume of the D_2_O solution of the host molecule.

***Note:*** The concentration of D_2_O solution of the host molecule is determined as the requirements of different stages of hierarchical self-assemblies.

11. The samples are prepared by step 9 and 10 according to the experimental requirements in different host/guest molar ratios from 1:0.1, 1:0.2, 1:0.3 … to 1:1.5 (Figure 2E).

**CRITICAL:** NMR tube may destroy the probe of NMR instrument once at wrong position in spinner, please be careful on the depth of NMR tube in spinner when putting them in (Figure 2C).

**CRITICAL:** NMR tube may scratch the skin when its broken, please be careful.

12. The parameter of NMR is set up as shown in Figure 2D through TopSpin software (More detail operating step please refer to Hilty et al.^2^).

***Note:*** The NMR instrument is very expensive. Please make sure you are qualified to operate it.

***Note:*** If the signal peak of the solvent D_2_O is much larger than signal peak of target compounds, suppress the signal peak of the solvent D_2_O.

***Optional:*** A detailed chemical structure of self-assembly can be obtained from 2D-NMR, such as correlation spectroscopy (COSY) and rotating-frame Overhauser effect spectroscopy (ROESY). (Ma et al.^3^)

13. All the NMR data in .fid files are imported into MestReNova to determine the variation tendency of hierarchical host-guest supramolecular self-assembly system, see Figure 2E.

### Scanning electron microscopy (SEM)

**Timing: 1.5–2 days**

This section describes SEM test for morphology studying of hierarchical host-guest supramolecular self-assemblies which have representative molar ratio of host/guest molecules. Please attention: this section is not suitable for very poor electrical conductive samples as well as magnetic samples.

14. The aqueous solution of the guest molecule is prepared according to the Part: Preparation of the aqueous solution of the guest molecule.
15. The aqueous solution of the host molecule is prepared according to the Part: Preparation of host molecule aqueous solutions or dispersions**.**
16. Mix the above-mentioned two aqueous solutions in different molar ratios to prepare representative hierarchical host-guest supramolecular self-assemblies which can be monitored by UV-vis and NMR experiments.

***Note:*** (1) All vessels should be washed with filtered deionized water. (2) The concentration of hierarchical host-guest supramolecular self-assemblies in water should be higher than the critical aggregate concentration (CAC, for acquiring CAC of sample, please refer to Yan et al.^4^) of each hierarchical host-guest supramolecular self-assembly to make sure that the photos of self-assemblies are easily taken. Meanwhile, the sample concentration does not need to exceed the critical concentration by an order of magnitude.

**Figure 3:**
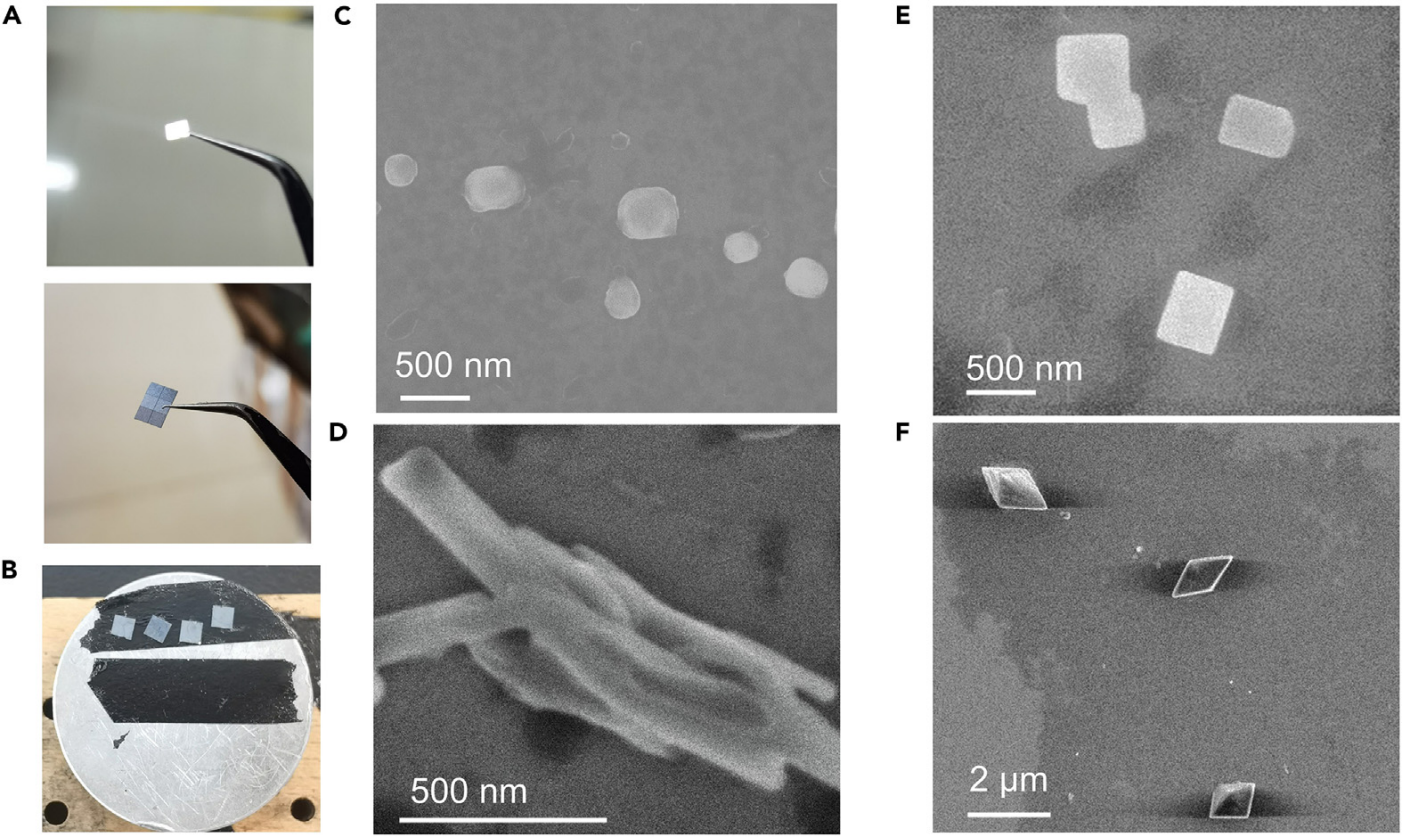
SEM test (A) The images for the front side (top) and the reverse side (bottom) of the wafer. The front side of the wafer possesses a smooth mirror face and the back side of the wafer possesses a rough surface. (B) The image of table for holding sample. In common, the sample is stuck on the table by black conductive tape. (C–F) SEM images for hierarchical host-guest supramolecular self-assemblies. The molar ratios of guest and host molecules are 1:0, 1:0.5, 1:1 and 1:1.5, respectively.

***Note:*** 1. The wafer should be washed by acetone and then dried. 2. The metal coating step is not recommended here. (If you need metal coating step for your sample preparation, please refer to Grillet et al.^5^)

17. The solution of hierarchical host-guest supramolecular self-assemblies should be left standing for more than 1 h before being dropped on a dry sliced wafer (Figure 3A).

**CRITICAL:** When handling the wafer, be careful of the pipette tip to avoid damaging the smooth surface of wafer.

18. Set the wafer with sample in the dark at a constant temperature (e.g., 25°C) for about one night.
19. Fix the wafer on the metal sample stage by double-sided conductive adhesive (Figure 3B).
20. Put the sample stage into specimen storehouse following with vacuuming.

***Note:*** The SEM instrument is very expensive. Please make sure you are qualified to operate it.

21. Find the self-assemblies from the sample in low magnification with original voltage carefully and patiently.

***Note:*** In this step, patience is very important.

22. Once you find the self-assemblies in proper morphology, please increase the magnification, turn down voltage (For example, 1.5–5 kV), focus on the self-assemblies, and take photos as soon as possible.

***Note:*** Don't stay at the same place for a long time to avoid destroying the sample by electron beam (Figure 6C).

23. Finally, we recommend adjusting and rearranging the photos in Microsoft PowerPoint for normalization (Figures 3C–3F).

***Note:*** The morphologies of the different hierarchical host-guest supramolecular self-assembly depend on the chemical structures of host and guest molecules. For example, the CPDB is an organic pyridinium salt, so the self-assembly morphology of CPDB tends to be spherical nanoparticles based on amphiphilic interaction. When the CPDBs interact with CB[8] molecules to form supramolecular self-assembly in different binding ratios, the rigid part of host-guest complexes increase, leading to the morphologies of the these supramolecular self-assemblies to be more and more angular and to form 1D worm-like nanoribbons, 2D rectangular nanoplates, and 3D micro polyhedrons. These morphologies have also been confirmed by SEM and further TEM test results.

### Transmission electron microscopy (TEM)

**Timing: 1.5–2 days**

This section describes TEM test for morphology studying of hierarchical host-guest supramolecular self-assemblies which possess representative molar ratio of host/guest molecules. Comparing to the SEM test, electrical conductivity of sample does not need to be concerned in TEM test. However, this section is not suitable for magnetic samples. Furthermore, as for sample preparation, the solvent evaporation shows little effect on the morphology of samples.

24. The preparation of the solution of hierarchical host-guest supramolecular self-assemblies is the same as that in the **Section: SEM test.**

***Note:*** (1) Attention again: All vessels should be washed with filtered deionized water. (2) For TEM test, the concentration of sample should be no higher than **8 × 10**^**–4**^**M.**

**Figure 4:**
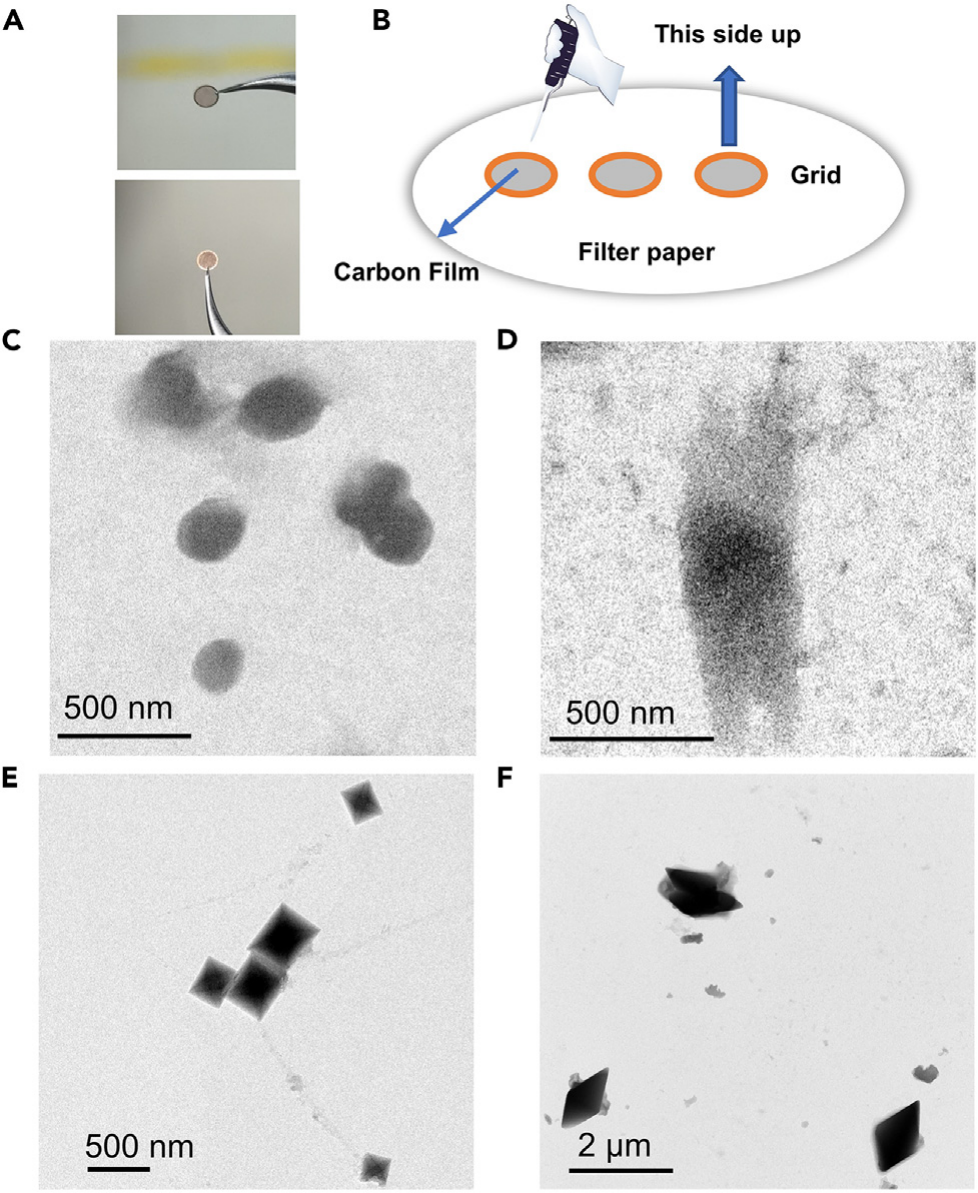
TEM test (A) The images of the front side (top) and the reverse side (bottom) of the TEM grid. The front side of TEM grid is covered by carbon film. (B) Illustration for preparing TEM sample. TEM grid is put on a filter paper with the front side up, followed by dropping sample on the TEM grid slowly. (C–F) The TEM images for hierarchical host-guest supramolecular self-assemblies. The molar ratios of guest and host are 1:0, 1:0.5, 1:1 and 1:1.5, respectively.

***Note:*** 1. Put a filter paper under the gilder TEM grids to keep the sample on the grids clean. 2. Normally, a drop (5–10 μL) of solution is enough for one gilder TEM grid. 3. Don’t mistake the front and back of the gilder TEM grid, that is, the side of gilder TEM grid with carbon film should be put face to you.

25. The solution of hierarchical host-guest supramolecular self-assemblies should be left standing for more than 1 h before being dropped into gilder TEM grids, see Figures 4A and 4B.

**CRITICAL:** When handling the grid, be careful of the pipette tips and the tweezers to avoid damaging the grid surface.

26. Set the gilder TEM grids with sample in the dark at a constant temperature (e.g., 25°C) for about one night.
27. The grid should be put into a standard TEM holder, followed by placing the TEM holder into specimen storehouse.

***Note:*** The TEM instrument (More detail about TEM instrument please refer to Wang et al.^6^) is very expensive. Please make sure you are qualified to operate it.

28. Find the self-assemblies from the sample in low multiplier lens carefully and patiently and take the photos in high multiplier lens.

***Note:*** In this step, patience is very important.

29. Finally, we recommend to adjust and rearrange the photos in Microsoft PowerPoint for normalization (Figures 4C–4F).

## Expected outcomes

Using this protocol, many hierarchical host-guest supramolecular self-assemblies can be analyzed, showing a multilevel state change of self-assembly. NMR and UV-vis spectra reveal the detailed structure variations of host-guest supramolecular complexation (Figures 1D, 1E, and 2E), and you may find and build the certain chemical architecture for each certain and appropriate hierarchical host-guest supramolecular self-assemblies if you are lucky. But you cannot determine a self-assembly model just relying on these spectra. Therefore, SEM and TEM images (Figures 3C–3F and 4C–4F) are the critical evidence to display the morphologies of these hierarchical host-guest supramolecular self-assembly directly. If possible, single crystal or stoichiometric calculation are also useful to reveal the construction model of the hierarchical host-guest supramolecular self-assemblies.

## Limitations

This protocol is broadly applicable to most samples of hierarchical host-guest supramolecular self-assembly system in the case of well-soluble samples. You may obtain beautiful research results from UV-vis spectra, TEM, SEM, and these tests can be operated in a relatively low concentration (∼10^–5^ M). However, NMR spectra requires higher concentration (at least 1 × 10^–3^ M) in D_2_O for clear peaks. The intermolecular structure and interactions between host and guest molecules can only be obtained from NMR results, which is the foundation to demonstrate the formation of hierarchical host-guest supramolecular self-assemblies.

**Figure 5:**
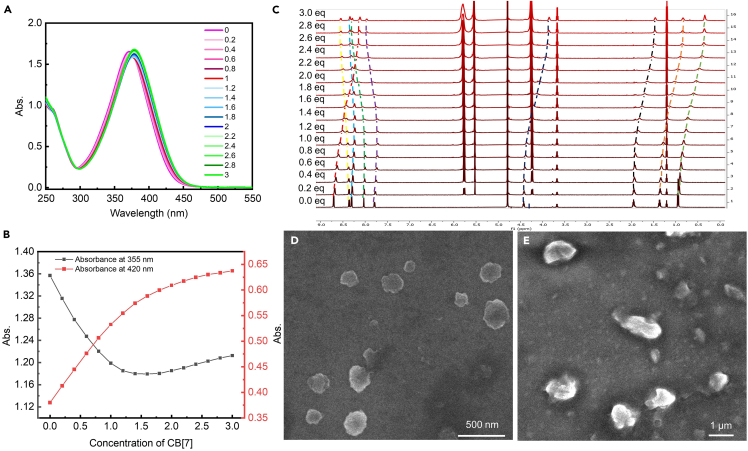
A case for CB[7]-based hierarchical host-guest supramolecular self-assemblies with irregular morphologies (A) UV-vis spectra of hierarchical host-guest supramolecular self-assemblies with CB[7] variation from 0 equiv. to 3.0 equiv. (B) The specific absorbance at 420 and 355 nm of hierarchical host-guest supramolecular self-assemblies with CB[7] variation from 0 equiv. to 3.0 equiv. (C) ^1^H-NMR spectra of hierarchical host-guest supramolecular self-assemblies with CB[7] variation from 0 equiv. to 3.0 equiv. The variation trends of the characteristic peaks of sample are marked by dashed lines with different colors. (D and E) SEM images for CB[7]-based hierarchical host-guest supramolecular self-assemblies with irregular morphology. The molar ratios of guest and host molecules are 1:1 and 1:2, respectively.

Another limitation is binding constant (K_a_) of host-guest supramolecular system. Low bonding constant leads to weak binding capability resulting in irregular morphology of assembly. The cucurbit[7]uril (CB[7]) is also used in Chen et al.,^1^ but the binding constant between CB[7] and guest molecules are much weaker than that between CB[8] and guest molecules. As shown in Figure 5, CB[7]-based self-assembly system with irregular morphology indicates that CB[7] is not a suitable building block for hierarchical self-assembly system in this work.

## Troubleshooting

### Problem 1

During titration, find UV-vis spectra of one solution sample of a group of spectra far deviates the trends of the titration, which belongs to invalid data. (Step 5 in UV-vis section)

### Potential solution

All sample solutions of titration should be prepared again for UV-vis spectra test instead of only replacing the invalid sample.

### Problem 2

The NMR peaks of sample are too weak to split clearly, while the concentration of sample cannot be increased because of the limit of solubility. (Step 11 and 12 in ^1^H-NMR section).

### Potential solution

Two ways for potential solution. First and more recommended: increase the scan times (Figure 2D). Because the signal-to-noise ratio of NMR is proportional to scan times, increasing the scan times will avoid influence from unstable topshim. Secondly (final attempt), decreasing D_2_O content of the mixed deuterium solvent to increase solubility for sample. But the D_2_O volume content should not be less than 90%.

### Problem 3

It is hard to distinguish the shifts of NMR peaks of guest molecules after adding host molecules (Step 13 in ^1^H-NMR section).

### Potential solution

Decrease the span of titration molar ratio for NMR test. For example, you can change the titration molar ratio from 1:0, 1:0.25, 1:0.5, 1: 0.75 and 1:1 to 1:0, 1:0.1, ……, 1:0.9 and 1:1.

### Problem 4

The background of SEM image or TEM possess unidentified pieces. (Step 21 in SEM section and Step 28 in TEM section)

### Potential solution

For both SEM and TEM tests, the unidentified pieces may come from: (1) unavoidable impurities from water and silicon wafer; (2) overhigh concentration or excess volume of sample solution dropped on the wafer. Thus, the steps in SEM or TEM parts should be carried out carefully to avoid artificial interference. Specially for SEM test, silicon wafer should be washed more than three times with ethanol or acetone carefully and dried under a constant temperature (e.g., 25°C), but there is no need of wash step for TEM grid. (Commercial TEM grid is clear. If you are still worried about introducing impurities, you can wash your tweezers.)

### Problem 5

It is hard to confirm morphology features of each hierarchical self-assembly state in SEM or TEM test. (Step 21 in SEM section and Step 28 in TEM section)

### Potential solution

**Figure 6:**
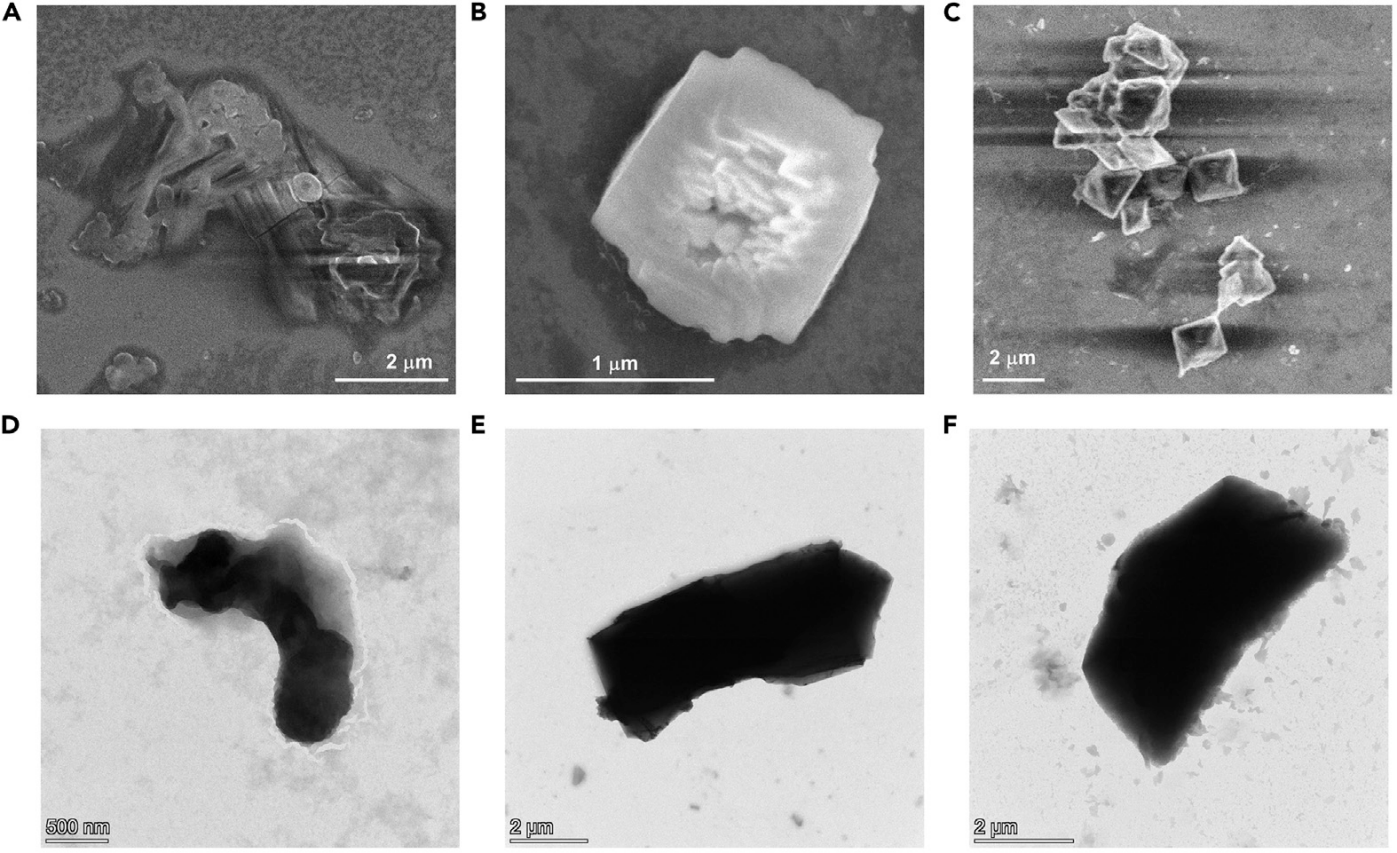
Some unsuccessful cases for SEM and TEM test for CB[8]-based hierarchical supramolecular self-assemblies (A–C) SEM images of incomplete supramolecular self-assemblies. The black traces and background are caused by prolonged electron beam shock. (D–F) TEM images of incomplete supramolecular self-assemblies.

Do the tests as much as you can. Because the non-covalent property of supramolecular self-assembly, the incomplete self-assemblies may happen in some samples occasionally. Find a morphology that appears most frequently which highly meets the expected assembling mechanism (Figure 6). Moreover, using energy dispersive analysis to determine the element contents of each self-assembly is also a direct approach for the determination of the required self-assemblies.

## Resource availability

### Lead contact

Further information and requests for resources and reagents should be directed to and will be fulfilled by the lead contact, Quan Li (quanli3273@gmail.com ).

### Materials availability

This study did not generate new unique materials.

## Data Availability

Raw datasets are available from the lead contact upon request. Processed datasets are provided in Chen et al.,^1^ 2022.
